# Quantitative DCE-MRI prediction of breast cancer recurrence following neoadjuvant chemotherapy: a preliminary study

**DOI:** 10.1186/s12880-022-00908-0

**Published:** 2022-10-20

**Authors:** Rajat Thawani, Lina Gao, Ajay Mohinani, Alina Tudorica, Xin Li, Zahi Mitri, Wei Huang

**Affiliations:** 1 Division of Hematology and Oncology, Knight Cancer Institute, Oregon Health & Science University, Sam Jackson Park Road, OCH14110, 97239 Portland, OR US; 2 Biostatistics Shared Resource, Knight Cancer Institute, Oregon Health & Science University, 3181 SW Sam Jackson Park Road, 97239 Portland, OR US; 3 Department of Internal Medicine, Oregon Health & Science University, 3181 SW Sam Jackson Park Road, 97239 Portland, OR US; 4 Department of Radiology, Oregon Health & Science University, 3181 SW Sam Jackson Park Road, 97239 Portland, OR US; 5 Advanced Imaging Research Center, Oregon Health & Science University, 3181 SW Sam Jackson Park Road, 97239 Portland, OR US

**Keywords:** Breast cancer, Dynamic contrast-enhanced (DCE) MRI, Recurrence, Neoadjuvant chemotherapy, Transfer rate constant (K^trans^)

## Abstract

**Introduction:**

Breast cancer patients treated with neoadjuvant chemotherapy (NACT) are at risk of recurrence depending on clinicopathological characteristics. This preliminary study aimed to investigate the predictive performances of quantitative dynamic contrast-enhanced (DCE) MRI parameters, alone and in combination with clinicopathological variables, for prediction of recurrence in patients treated with NACT.

**Methods:**

Forty-seven patients underwent pre- and post-NACT MRI exams including high spatiotemporal resolution DCE-MRI. The Shutter-Speed model was employed to perform pharmacokinetic analysis of the DCE-MRI data and estimate the K^trans^, v_e_, k_ep_, and τ_i_ parameters. Univariable logistic regression was used to assess predictive accuracy for recurrence for each MRI metric, while Firth logistic regression was used to evaluate predictive performances for models with multi-clinicopathological variables and in combination with a single MRI metric or the first principal components of all MRI metrics.

**Results:**

Pre- and post-NACT DCE-MRI parameters performed better than tumor size measurement in prediction of recurrence, whether alone or in combination with clinicopathological variables. Combining post-NACT K^trans^ with residual cancer burden and age showed the best improvement in predictive performance with ROC AUC = 0.965.

**Conclusion:**

Accurate prediction of recurrence pre- and/or post-NACT through integration of imaging markers and clinicopathological variables may help improve clinical decision making in adjusting NACT and/or adjuvant treatment regimens to reduce the risk of recurrence and improve survival outcome.

## Introduction

Breast cancer is the most commonly diagnosed cancer in women, and the second most common cause of death [1]. Locally advanced breast cancer is a subset where patients have either stage IIB disease (T3N0) or stage IIIA to IIIC disease. Neoadjuvant chemotherapy (NACT) is being utilized as the standard of care for treatment of this subset of patients [2]. The main objectives of NACT is to downstage the malignancy to make the cancer amenable to breast conservation surgery, as well as real-time evaluation of response to NACT to guide adjuvant treatment decisions. Many variables assessing response to NACT have been linked to prognosis, including residual cancer burden (RCB) [3], breast cancer subtype [4], clinical and pathological staging[5], and estrogen receptor status and tumor grade [6].

Patients who achieve pathological complete response (pCR) exhibit excellent prognosis. This is, however, not the case for most patients who have residual disease following NACT [7]. Measurement of residual breast cancer burden using RCB index has shown to be able to predict distant recurrence free survival after NACT, patients with RCB II or higher being at higher risk of these recurrences [3]. But by itself, RCB is an unreliable marker of recurrence and there is a need for a better biomarker. For example, data from the CREATE-X clinical trial show that 74% of patients with Human Epidermal growth factor Receptor 2 (Her2)-negative residual disease (many with RCB II or III) were alive and free from recurrence at three years without any adjuvant treatment [8]. In a study of Her2-positive patients, the KATHERINE trial showed that 77% of patients with residual disease were disease free after 3 years with continued adjuvant trastuzumab therapy [9]. Accurate identification of patients at high risk of recurrence post NACT may help select for appropriate treatment escalation and de-escalation strategies to improve outcomes. Current risk assessment models such as RCB rely on knowledge of surgical pathology following NACT, and do not offer an opportunity to adjust or escalate therapy in the neoadjuvant setting. This is where early prediction of treatment resistance and subsequently risk of recurrence using imaging combined with clinicopathologic variables can help guide treatment decisions, including evaluation of novel therapies aimed to augment response and improve patient outcomes.

Multiple studies have been conducted to predict pathological complete response in patients undergoing NACT using tumor tissue, blood or imaging biomarkers [10–12]. Quantitative imaging biomarkers derived from pharmacokinetic (PK) analysis of dynamic contrast-enhanced magnetic resonance imaging (DCE-MRI) data have been shown capable of predicting pathologic response of breast cancer to NACT after only one or two NACT cycles [13–16]. Quantitative DCE-MRI not only measures tumor morphology such as tumor size and shape, but also estimates quantitative parameters related to the physiologies of microvasculature and perivascular tissue, which include perfusion and permeability, and extravascular and extracellular volume fraction [17, 18]. In response to cancer therapies, changes in these quantitative functional imaging biomarkers are often found to precede any observable changes in tumor size [13, 19–21].

A few breast DCE-MRI studies demonstrated prediction of survival following NACT using quantitative [22] or semi-quantitative imaging biomarkers [23]. However, few DCE-MRI studies have reported prediction of breast cancer recurrence following NACT and none of them used quantitative DCE-MRI parameters [24–26]. In this preliminary study, by retrospectively correlating post-NACT breast cancer recurrence outcomes with pre- and post-NACT quantitative DCE-MRI parameters and MRI tumor size measurements, we sought to investigate the predictive performances of these MRI metrics, alone and in combination with clinicopathological variables, for prediction of recurrence in patients treated with NACT.

## Methods

### Patient cohorts

Data collection for this study was conducted under the institutional review board approved protocol 5492 at Oregon Health & Science University (Portland, Oregon, U.S.A.). All patients who were diagnosed with grade 2–3 invasive breast tumors and scheduled to undergo NACT as standard of care were eligible for the study. An informed consent was obtained for participation in a longitudinal study using a research MRI protocol, which was conducted from 2012 to 2016 and included four MRI sessions: before NACT (visit 1 (V1)), after the first but before the second NACT cycle (V2), at midpoint of NACT (V3; before the change of NACT drugs), and after completing the entire NACT course but prior to surgery (V4), respectively. Clinicopathological variables collected for study participants included patient demographics, chemotherapy regimens, tumor histology, grade, stage, receptor status (estrogen receptor (ER), progesterone receptor (PR) and HER2), and nodal status. Pathologic response to NACT and RCB index (0, I, II, and III with RCB = 0 equivalent to pCR) were determined from the post-NACT resection specimens. The recurrence and survival outcome data were collected in September 2021 from the electronic medical records.

The forty-seven patients who consented to this study received standard of care NACT with taxanes and anthracyclines, in addition to trastuzumab for those with HER2-positive malignancy. Three of the 47 patients were enrolled in the NACT ISPY-2 trial [27], where they received standard of care regimen in addition to an experimental drug. As a requirement for participation in the ISPY-2 trial, this subset of patients also underwent a separate ISPY-2 MRI protocol, which included a DCE-MRI scan with much lower temporal resolution (80–100 s) compared to the DCE-MRI scan used in this research study (see data acquisition details below), at the same four time points. For these three patients, there was an interval of at least 24 h between this study’s research MRI and the ISPY-2 MRI studies to allow gadolinium-based contrast agent clearance from the body. The DCE-MRI results from the first 28 patients of this study cohort were previously reported [13] to demonstrate the capabilities of quantitative DCE-MRI PK parameters for early prediction and evaluation of breast cancer pathologic response to NACT.

### DCE-MRI data acquisition

All the breast MRI scan sessions were performed using a 3 T Siemens Tim Trio system. The built-in body coil was used as the RF transmitter and a four-channel bilateral phased-array breast coil was used as the receiver. In each MRI session, pilot scans and axial T_2_-weighted MRI with fat-saturation and axial T_1_-weighted MRI without fat-saturation were performed before axial DCE-MRI with bilateral full breast coverage. The DCE-MRI images with fat-saturation (using the method of water excitation only) were acquired using a 3D gradient echo TWIST (Time-resolved angiography WIth Stochastic Trajectories) sequence, which adopts a scheme of k-space undersampling during acquisition and view sharing during reconstruction to achieve high temporal and spatial resolution simultaneously. [28] The acquisition parameters included: 10^o^ flip angle, TE/TR = 2.9/6.2 ms, parallel imaging factor (iPAT) of 2, 30–34 cm FOV, 320 × 320 resolution in both read and phase directions, and 1.4 mm slice thickness. The DCE-MRI scan time was limited to about 10 min. Due to differences in patient breast size, the number of image slices in each DCE frame varied from 96 to 128, resulting in 14–20 s temporal resolution and 28–38 frames. The contrast agent, Gd(HP-DO3A) [ProHance (Bracco Diagnostic Inc.)], was injected intravenously at a dose of 0.1 mmol/kg and a rate of 2 mL/s by a programmable power injector at the beginning of the third DCE frame acquisition, followed by a 20-mL saline flush at the same injection rate. For the purpose of quantifying native tissue T_1_, T_10_, proton density-weighted images spatially co-registered with the DCE-MRI images were acquired immediately before the DCE scan [28, 29], with the same acquisition sequence and parameters except for 5° flip angle and TR = 50 ms.

### DCE-MRI data analysis

Using post-contrast DCE images at approximately 120–150 s after the contrast injection, the breast tumor boundaries were manually delineated by experienced radiologists on all image slices containing the contrast-enhanced tumor, generating tumor regions of interest (ROIs) for quantitative analysis. The longest diameter (LD) of the tumor was measured by the same radiologists based on the RECIST (Response Evaluation Criteria in Solid Tumors) guidelines [30, 31].

The DCE-MRI time-course data in each voxel within the tumor ROIs were subjected to PK analysis using a home-built Matlab software package based on the Shutter-Speed PK model [13, 32, 33] which takes into account the intercompartmental water exchange kinetics. The fast-exchange-regime version [13, 33] of the Shutter-Speed model was used to fit the DCE data in this study to extract the following three parameters: K^trans^ (transfer rate constant), v_e_ (extravascular and extracellular volume fraction), and τ_i_ (mean intracellular water lifetime). τ_i_ is unique to the Shutter-Speed model and is used to account for cross-cell membrane water exchange kinetics in the model. The efflux rate constant, k_ep_, was calculated as K^trans^/v_e_. The tumor mean PK parameter value was calculated by averaging all the voxel parameter values. The detailed formulations used for PK analysis of the DCE-MRI data collected in this study are shown in Tudorica et al. [13].

Arterial input function (AIF) and T_10_ are usually required in PK analysis of DCE-MRI data. For this study, an average AIF from individually measured AIFs (from an axillary artery) in a separate breast DCE-MRI study [34] [with sagittal single-breast coverage and higher temporal resolution (< 10 s)] was used for PK analysis. It has been shown that it is a reasonable approach to use a population-based mean AIF for PK analyses of DCE-MRI data from the same tissue of interest if the contrast agent, dose, injection rate, and injection site are kept the same [35], which is the case for this study in relation to the separate study [33]. Using the equation for steady-state signal from a spoiled gradient-echo sequence [13, 28], the voxel T_10_ values within the tumor ROIs were calculated by comparing the voxel signal intensities between the images in the second baseline DCE frame and the spatially co-registered proton density images.

Six of the 12 patients who achieved pCR show no visible tumor contrast enhancement at V4. For the purpose of PK analysis of V4 DCE-MRI data in these 6 cases, the radiologists copied three tumor ROIs on three consecutive image slices through the central portion of the tumor from V3 DCE-MRI, where tumor contrast enhancement was clearly visible in all the cases, and pasted onto three consecutive slices in V4 DCE-MRI based on assessment of anatomic similarities between V3 and V4 MRI. Since the low temporal resolution and small number of time frames of the ISPY-2 DCE-MRI data are not suitable for accurate PK data analysis, the Shutter-Speed model analysis was not applied to the additional ISPY-2 DCE-MRI data from the three patients enrolled in the ISPY-2 trial.

### Statistical analysis

Appropriate descriptive statistics were used to summarize the patient clinicopathological and MRI characteristics for the study cohort by recurrence status. Means and standard deviations were reported for continuous variables and proportions were reported for categorical variables. To align with the current standard clinical management of breast cancer patients treated with NACT, where usually only pre- and post-NACT MRI exams are prescribed, only the V1 and V4 MRI metrics from this study, as well as the corresponding percent changes (V4 relative to V1, V4_1%), were included in statistical analysis. To compare means of continuous variables between the recurrence and non-recurrence groups, independent t-test assuming equal variance was used, as there is no prior evidence from studies of similar patient cohorts with sufficient sample size in the literature to assume that the two groups have different variance in MRI characteristics. Fisher’s exact test was used to compare the distribution of categorical variables between the groups. Univariable logistic regression (ULR) C statistics, also known as the area under the receiver operating characteristic (ROC) curve (ROC AUC), for discrimination of the recurrence and non-recurrence groups was reported for each MRI metric. 95% confidence interval (CI) for AUC was calculated using the DeLong method [36].

In building multivariable models for recurrence prediction, Firth logistic regression was used to mitigate potential bias caused by rare events and accommodate quasi-complete separation in the data [37, 38]. Clinicopathological variables with a p-value < 0.2 in the univariate analysis, which were RCB, stage, and age, were used to build the initial multivariable Firth logistic regression model. Next, an automated stepwise (backwards) model selection procedure by AIC (Akaike information criterion) was used to select a parsimonious main effect only model that accomplishes a desired level of prediction without over-fitting the current data. This final clinicopathological variable-only model included RCB and age. Based on this model, at the optimal cutoff point determined by Youden’s Index, which equivalently maximizes the sum of sensitivity and specificity, prediction performance measures with 95% exact confidence interval were calculated [39]. Next, in this model, MRI metrics were added one at a time to assess added value of a single MRI metric in prediction of recurrence. In such models, Wald p-values were calculated to evaluate the significance of contribution by the MRI metric to the prediction of recurrence. ROC AUC was used to examine predictive performance for these models, whether including RCB and age only or including RCB, age, and one MRI metric. Repeated 5-fold cross validation was performed to obtain mean cross-validated (cv) ROC AUC.

In addition, the added value of multiple MRI metrics in predictive performance was investigated. Since many MRI metrics were highly correlated such as K^trans^ and k_ep_, principal components (PCs) of all MRI metrics at V1 and V4, as well as V4_1%, were calculated to capture variances in fewer dimensions. With the aid of PC scree plots, adding only the first PC (PC1) to RCB and age in the predictive model was determined to be the optimal approach to avoid severe over-fitting by adding multiple PCs. ROC AUCs from the MRI metric-enhanced models (with a single metric or PC1) were compared to that from the clinicopathological variable-only model (RCB and age) using the DeLong test based on U-statistics theory and asymptotic normality for paired ROC curves [36]. The calculated p-values are nominal p-values without multiple testing adjustments.

All statistical analysis was performed using R version 4.1.1 [40].

## Results

Table 1 shows the clinicopathological data of all the patients who were enrolled in this study. Of these 47 patients, seven experienced a recurrence with a median time to recurrence of 5 months, whether locoregional or distant. The distributions of these two subgroups were similar in terms of tumor type and receptor status. As described below, age and RCB Index were different between the groups and predicted recurrence. The follow up time was longer for patients without recurrence, as they were more likely to be alive and in follow up.

**Table 1:** Clinicopathological data of enrolled participants (N = 47)

Clinicopathological Variables	Entire cohort (N = 47)	Non-recurrence (N = 40)	Recurrence (N = 7)
Age (Mean ± SD)	47.6 ± 12.9 years	48.6 ± 13.3 years	39.7 ± 8.2 years
Follow up (Median, IQR)	80, 33.5 months	85.5, 44.4 months	27.1, 41.4 months
Tumor type (N [%]) IDC ILC IMC	42 (89.3%) 2 (4.3%) 3 (6.4%)	36 (90%) 2 (5%) 2 (5%)	6 (85.7%) 0 1 (14.3%)
Breast Cancer subtype (N [%]) Hormone Receptor Positive (ER or PR)	27 (57.4%)	23 (57.5%)	4 (57.1%)
HER2-Receptor Positive	24 (51.1%)	21 (52.5%)	3 (42.9%)
Triple Negative Receptor	8 (17.0%)	7 (17.5%)	1 (14.3%)
RCB Index (N [%]) pCR Class I Class II Class III	12 (25.5%) 10 (21.3%) 18 (38.3%) 7 (14.9%)	12 (30%) 9 (22.5%) 16 (40%) 3 (7.5%)	0 1 (14.3%) 2 (28.6%) 4 (57.1%)
Recurrence (N [%])	7 (14.9%)		

IQR – Interquartile range, IDC-Invasive Ductal Carcinoma, ILC- Invasive Lobular Carcinoma, IMC- Invasive Mammary Carcinoma, ER – Estrogen Receptor, PR – Progesterone Receptor, HER2 – Human Epidermal growth factor Receptor 2, RCB – Residual Cancer Burden, pCR – Pathological Complete Response

### Prediction of recurrence with a single MRI metric

**Fig. 1:**
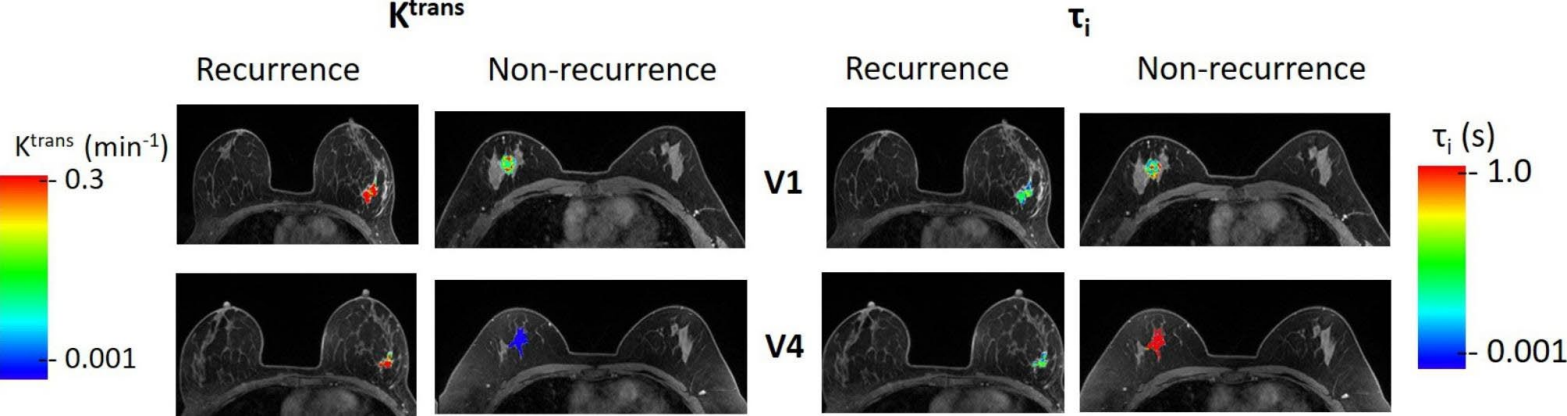
Breast tumor color K^trans^ (left panels) and τ_i_ (right panels) parametric maps from a patient with recurrence (48 years old, grade 3 invasive ductal carcinoma, ER-, PR+, HER2-, RCB = III) following NACT and a patient without recurrence (39 years old, grade 2 invasive ductal carcinoma, ER+, PR+, HER2+, RCB = 0 (pathologic complete response)). The color maps are overlaid onto the post-contrast DCE-MRI image slices that were through the centers of the two tumors, respectively. The maps in the top row were obtained from the DCE-MRI data collected pre-NACT (at V1), while those in the bottom row were obtained post-NACT (at V4). The color scales of K^trans^ and τ_i_ are kept the same, respectively, for both patients and both MRI visits for comparison purposes. This figure is intended to show voxel-based quantitative DCE-MRI parameter values pre- and post-NACT. The images displayed for the two patients are cropped and zoomed at different scales, and therefore, should not be used to compare tumor size between the patients

**Fig. 2:**
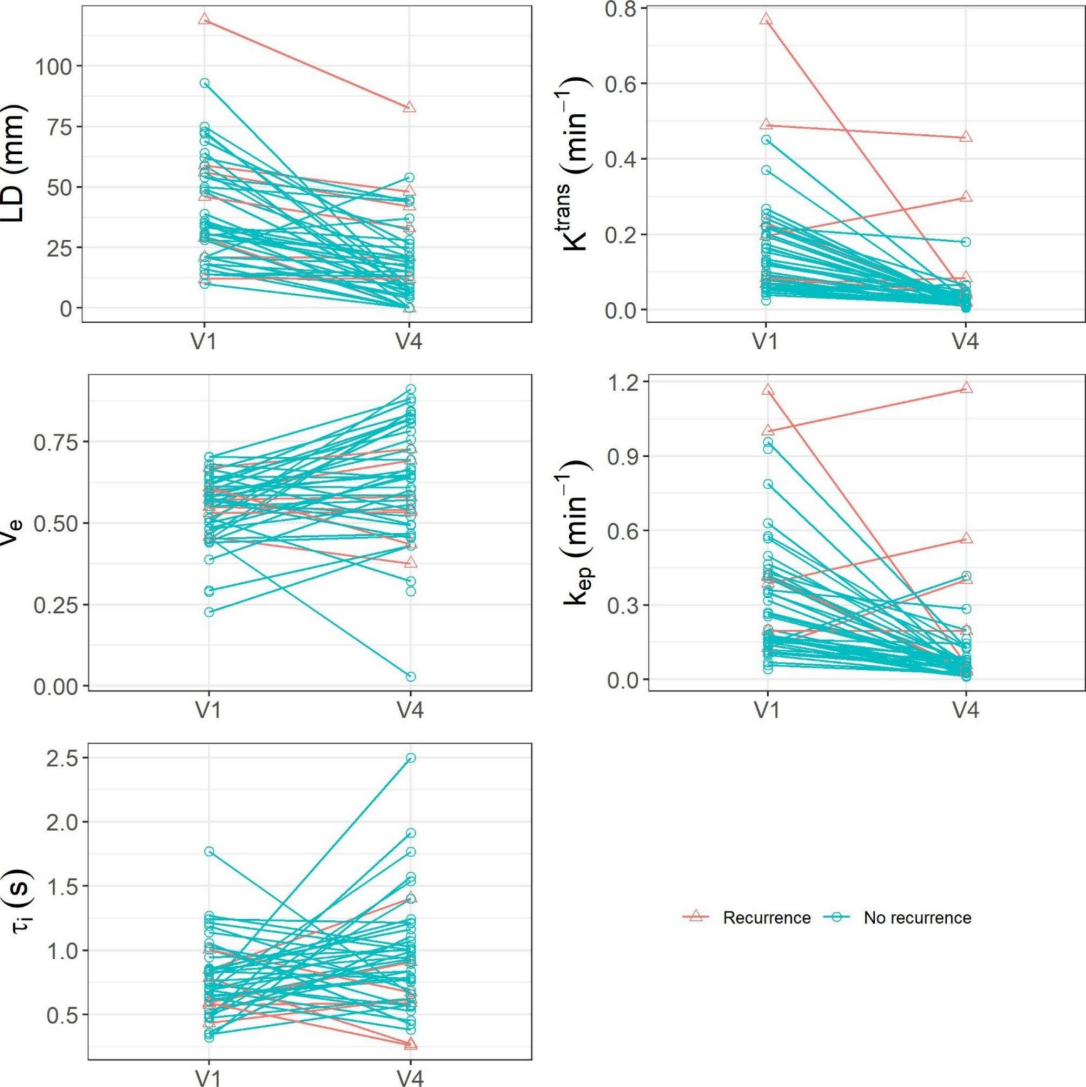
Scatter plots of tumor LD and mean tumor K^trans^, v_e_, k_ep_, and τ_i_ values for all the individual patients with (red triangle) and without (blue circle) recurrence. The data points at V1 and V4 are connected with solid lines of corresponding colors

Table 2 shows the mean ± SD values of the breast tumor MRI metrics, including LD and DCE-MRI parameters, for the recurrence and non-recurrent groups, the t-test p-values in comparing the two groups, as well as the ULR C statistics values for prediction of recurrence vs. non-recurrence. At V1 (pre-NACT), only K^trans^ and k_ep_ showed significant (p < 0.05) differences between the two groups of patients; at V4 (post-NACT), K^trans^, k_ep_, and LD exhibited significant (p < 0.05) differences with the difference in τ_i_ approaching statistical significance (p = 0.083). For V4_1% of the MRI metrics, again, only K^trans^ and k_ep_ demonstrated significant (p < 0.05) differences. For prediction of recurrence, only V1 K^trans^ and V4 LD, K^trans^, k_ep_, and τ_i_ showed a ROC AUC > 0.7 with V4 K^trans^ having the largest AUC of 0.812. As an example, Fig. 1 shows the voxel-based color tumor K^trans^ (left panels) and τ_i_ (right panels) parametric maps on a single image slice for a patient with breast cancer recurrence (48 years old, grade 3 invasive ductal carcinoma, ER-, PR+, HER2-, RCB = III) and a patient without recurrence (39 years old, grade 2 invasive ductal carcinoma, ER+, PR+, HER2+, RCB = 0 (pathologic complete response)) at V1 (top row) and V4 (bottom row). The color scales for K^trans^ and τ_i_ are kept the same, respectively, for both patients and both MRI visits for the purpose of comparison. The patient with recurrence showed substantially high K^trans^ values in the tumor compared to the patient without recurrence at V1, and the difference in K^trans^ between the two tumors became even more prominent at V4. On the other hand, the difference in tumor τ_i_ between the two patients was not clearly observable at V1, with the patient without recurrence showing slightly higher τ_i_. However, at V4, the patient without recurrence showed substantially higher tumor τ_i_ compared to the patient with recurrence. For both DCE-MRI parameters, the patient without recurrence showed substantially larger changes from V1 to V4 (decreases in K^trans^ and increases in τ_i_) compared to the patient with recurrence. Figure 2 shows plots of LD and mean tumor K^trans^, v_e_, k_ep_, and τ_i_ values for all the individual patients at V1 and V4. It is important to note that mean tumor values of the DCE-MRI parameters are reported in Fig. 2, while the parametric maps in Fig. 1 are shown for only one image slice through the center of the tumor.

**Table 2:** MRI Metrics for Prediction of Recurrence

MRI metrics	No recurrence (N = 40) *Mean (SD)*	Recurrence (N = 7) *Mean (SD)*	p-value	ULR C statistics value	95% CI
**RECIST tumor size measurement**					
LD_V1 (mm)	38.73 (20.37)	48.83 (35.64)	0.29	0.541	[0.26, 0.822]
LD_V4 (mm)	16.23 (14.28)	34.09 (27.18)	0.013	0.714	[0.459, 0.97]
LD_V4_1%	-0.58 (0.33)	-0.36 (0.36)	0.12	0.684	[0.43, 0.938]
**DCE-MRI parameters**					
K^trans^_V1 (min^− 1^)	0.14 (0.10)	0.30 (0.25)	0.007	0.725	[0.501, 0.95]
v_e__V1	0.54 (0.11)	0.57 (0.07)	0.5	0.553	[0.349, 0.757]
k_ep__V1 (min^− 1^)	0.31 (0.23)	0.53 (0.40)	0.047	0.681	[0.45, 0.913]
τ_i__V1 (s)	0.78 (0.30)	0.69 (0.20)	0.45	0.593	[0.375, 0.812]
K^trans^_V4 (min^− 1^)	0.03 (0.03)	0.14 (0.17)	< 0.001	0.812	[0.636, 0.988]
v_e__V4	0.63 (0.19)	0.56 (0.13)	0.347	0.647	[0.445, 0.848]
k_ep__V4 (min^− 1^)	0.08 (0.08)	0.35 (0.41)	< 0.001	0.752	[0.530, 0.974]
τ_i__V4 (s)	0.99 (0.44)	0.67 (0.39)	0.083	0.726	[0.489, 0.962]
K^trans^_V4_1%	-0.74 (0.24)	-0.37 (0.60)	0.006	0.62	[0.288, 0.953]
v_e__V4_1%	0.18 (0.38)	-0.02 (0.16)	0.175	0.699	[0.537, 0.861]
k_ep__V4_1%	-0.66 (0.48)	0.00 (1.13)	0.012	0.628	[0.295, 0.961]
τ_i__V4_1%	0.53 (1.07)	0.02 (0.50)	0.223	0.617	[0.389, 0.844]

ULR - Univariable logistic regression, 95% CI – 95% confidence interval for the C value, LD – Longest diameter, V1 – Visit 1 (pre-NACT MRI), V4 – Visit 4 (post NACT, pre-surgery MRI), V4_1% - corresponding percent changes (V4 relative to V1)

We also examined the association between time to recurrence from time of surgery and each MRI metric using univariable Cox PH model. Statistical significance of the results from this alternative approach, reflected in the p values (data not shown), is consistent with that from t-test, as shown in Table 2.

### Prediction of recurrence with multi-clinicopathological variables

As described above, the parsimonious multivariable Firth logistic regression model for prediction of recurrence using the clinicopathological variables contained RCB and age only. The ROC AUC of this combination was 0.900 (95% CI [0.762, 1]) (Table 3). At the optimal cutoff point determined by Youden’s index, the sensitivity was 0.86 (95% CI [0.42-1.00]), specificity was 0.89 (95% CI [0.75–0.97]) and the positive predictive value was 0.60 (95% CI [0.35–0.99]).

**Table 3:** Predictive Performances for Recurrence Using Clinicopathological Variables Only and Combinations of Clinicopathological Variables and MRI Metrics

Model	Wald test p-	value	ROC AUC	95% CI	cv ROC AUC
**Clinicopathological variables**					
RCB + Age	N/A		0.900	[0.762, 1]	0.797
**Clinicopathological variables and individual MRI metrics**					
LD_V1	0.807		0.892	[0.747, 1]	0.744
LD_V4	0.537		0.900	[0.760, 1]	0.769
LD_V4_1%	0.647		0.900	[0.778, 1]	0.730
K^trans^_V1	0.116		0.946	[0.878, 1]	0.798
v_e__V1	0.659		0.907	[0.795, 1]	0.777
k_ep__V1	0.137		0.942	[0.872, 1]	0.755
τ_i__V1	0.486		0.911	[0.797, 1]	0.754
K^trans^_V4	0.081		0.965	[0.916, 1]	0.814
v_e__V4	0.441		0.919	[0.808, 1]	0.814
k_ep__V4	0.071		0.961	[0.908, 1]	0.845
τ_i__V4	0.367		0.911	[0.812, 1]	0.725
K^trans^_V4_1%	0.296		0.938	[0.862, 1]	0.781
v_e__V4_1%	0.289		0.927	[0.824, 1]	0.843
k_ep__V4_1%	0.582		0.911	[0.799, 1]	0.782
τ_i__V4_1%	0.668		0.892	[0.770, 1]	0.720
**Clinicopathological variables and Principal Components of all MRI metrics**					
PC1	0.071		0.961	[0.910, 1]	0.837

ROC AUC - area under the receiver operating characteristic curve, 95% CI – 95% confidence interval for ROC AUC, cv – cross validated, N/A – not applicable, LD – Longest diameter, V1 – Visit 1 (pre-NACT MRI), V4 – Visit 4 (post NACT, pre-surgery MRI), V4_1% - corresponding percent changes (V4 relative to V1), PC1 – first principal components of all MRI metrics

### Added value of MRI metrics in prediction of recurrence

**Fig. 3:**
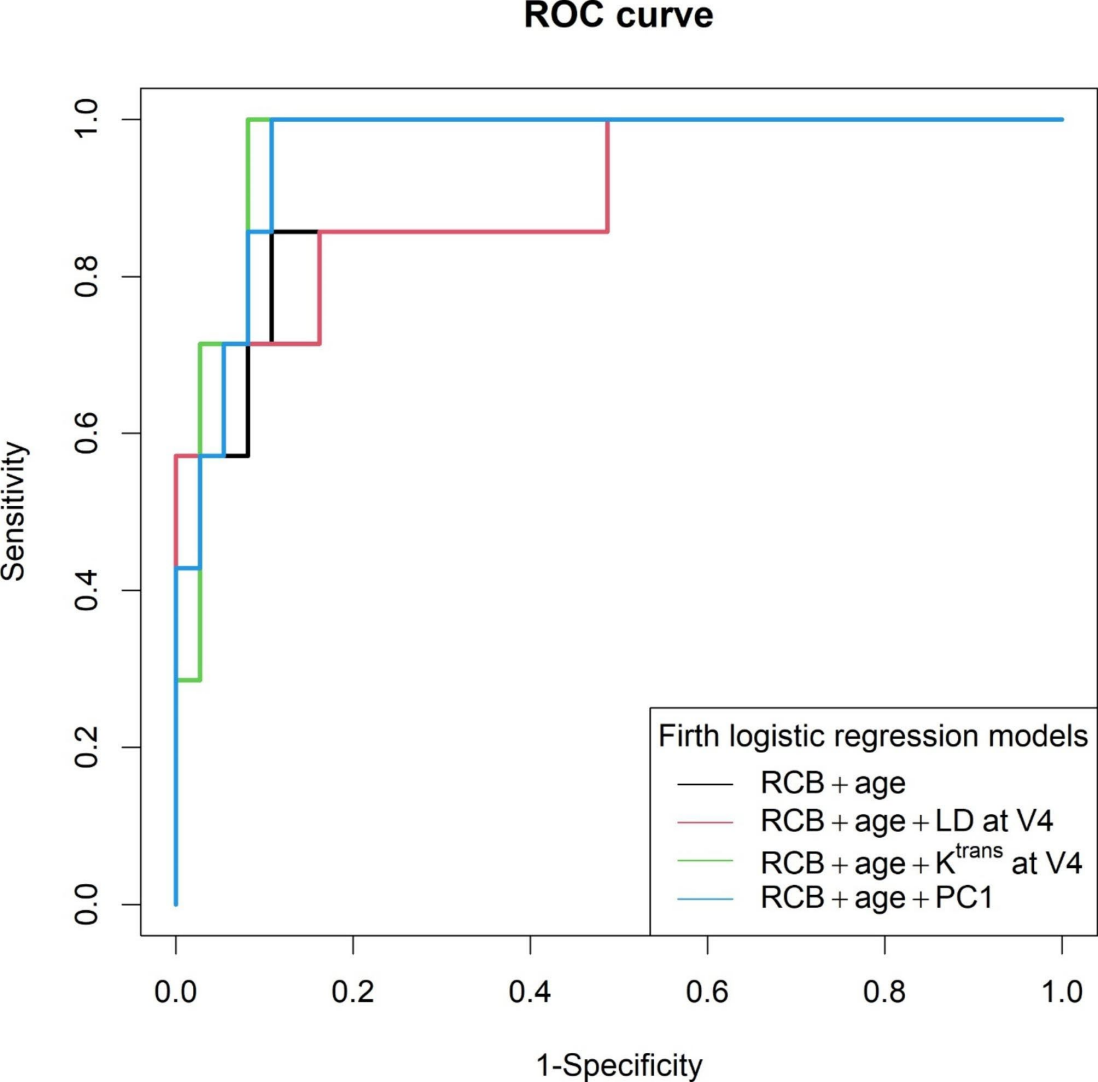
Empirical ROC curves for prediction of recurrence vs. non-recurrence: RCB and age only (black, AUC = 0.900), RCB and age and LD at V4 (red, AUC = 0.900), RCB and age and K^trans^ at V4 (green, AUC = 0.965), and RCB and age and PC1 of all MRI metrics (blue, AUC = 0.961), respectively

Table 3 shows ROC AUC (with 95% CI) and cv ROC AUC values for prediction of recurrence vs. non-recurrence when using clinicopathological variables of RCB and age only, combining RCB and age with a single MRI metric, and combining RCB and age with PC1 of all MRI metrics (including V1, V4, and V4_1%). The listed Wald test p-values indicate the significance of adding a single MRI metric or PC1 of all MRI metrics (to RCB and age) in prediction of recurrence. The addition of V1, V4, or V4_1% LD to RCB and age in the model did not improve the predictive performance with AUCs ≤ 0.900. However, with the exception of V4_1% of τ_i_, the addition of each single quantitative DCE-MRI parameter or PC1 of all parameters, improved predictive accuracy with AUCs > 0.900. The addition of V4 K^trans^ to RCB and age showed the largest improvement in predictive accuracy from AUC = 0.900 to AUC = 0.965. It is interesting to note that, at V1 (pre-NACT), the addition of K^trans^ alone improved AUC from 0.900 to 0.946. As an example, Fig. 3 shows the ROC curves for models including RCB and age only (black), RCB and age and V4 LD (red), RCB and age and V4 K^trans^ (green), and RCB and age and PC1 (blue), respectively. The Wald test shows that the additional contributions of V4 K^trans^, V4 k_ep_, and PC1 (on top of RCB and age) in prediction of recurrence were approaching statistical significance, with p = 0.081, 0.071, and 0.071, respectively. However, the increases in ROC AUC values were not statistically significant: e.g., p = 0.371 when comparing AUC of RCB and age and PC1 with that of RCB and age only.

## Discussion

The preliminary results from this study of 47 patients show the potential of added value of MRI metrics when combined with clinicopathological data in prediction of breast cancer recurrence in patients who underwent NACT. None of any single MRI metric, LD or quantitative DCE-MRI parameters pre- or post-NACT, was as accurate as the combined clinicopathological variables of RCB and age in prediction of recurrence. However, when a single quantitative DCE-MRI parameter or PC1 of all MRI metrics was added to RCB and age in the predictive model, this integration of imaging and clinicopathological characteristics seemed to perform better than the approach of clinicopathological variables only in prediction of recurrence. The increases in predictive performances following the addition of MRI metrics were not statistically significant. This may be partially due to potentially less reliable AUC estimates in our small sample size and inflated AUC values due to class imbalance between the recurrence (N = 7) and non-recurrence (N = 40) groups [41]. It is worth noting that, while the addition of functional and quantitative DCE-MRI parameters to clinicopathological variables improved predictive performance for recurrence, the addition of RECIST-based tumor LD at V1, V4 or V4_1% showed no positive effects.

The results from this preliminary study suggest that the addition of quantitative DCE-MRI parameters to clinicopathological variables can potentially help risk-stratify patients who are at a higher risk of recurrence, as it confers a poor prognosis in these patients with 5-year overall survival in only 58–71% of them [42]. The level of predictive accuracy for recurrence demonstrated in this study can help identify the exact patients who will benefit from addition of adjuvant treatment to prevent recurrence after NACT and surgery. Importantly, the RCB information is only available after NACT and surgery, precluding the ability to augment neoadjuvant therapy approaches, which is currently an active area of investigation. Ongoing trials such as the ISPY-2 study are evaluating novel designs to predict RCB based on clinical including imaging criteria prior to surgical resection. This would allow patients predicted to have residual disease the opportunity to enroll and receive novel therapies, including investigational targeted and immune therapies, in an attempt to improve pathologic responses. It is in this setting that a robust imaging biomarker can be extremely useful to help accurately identify these patients, and spare ones predicted to have a complete response added toxicity. In our study, the predictive accuracy for recurrence by the K^trans^ parameter alone before NACT (ULR C = 0.725 in this study) may potentially help guide the clinicians towards intensifying NACT regimens to improve pCR in certain cases, while de-escalating in others to avoid overtreatment.

There are only a few other studies that used DCE-MRI to predict recurrence in similar groups of patients, which performed radiomics analysis of DCE images rather than extracting quantitative functional parameters [26, 43]. This preliminary study shows that, similar to the case of predicting pathologic complete response to NACT [13–16], quantitative DCE-MRI parameters that measure breast tumor biological properties are superior to tumor size measurement in prediction of breast cancer recurrence following NACT, whether alone or in combination with clinicopathological variables. As measures of perfusion and permeability, both pre- and post-NACT tumor K^trans^ and k_ep_ tended to be higher in patients with recurrence compared to those without recurrence, suggesting high perfusion and permeability is associated with high risk for recurrence. Several studies [44–47] have demonstrated that the τ_i_ parameter, which is exclusive to the Shutter-Speed PK model, is an imaging biomarker of tissue metabolic activity with an inverse relationship. There was no clear difference in pre-NACT τ_i_ between patients with and without recurrence. For post-NACT τ_i_, although not statistically significant, the patients with recurrence tended to have lower post-NACT τ_i_, or higher metabolic activity, compared to those without recurrence, suggesting that risk of recurrence could be high if the tumor metabolic activity remains high after NACT treatment. The approach of PC analysis of all the MRI metrics is equivalent to a multi-parametric approach of combining the tumor size and DCE-MRI parameters of perfusion and permeability (K^trans^ and k_ep_), metabolic activity (τ_i_), and interstitial space (v_e_). It is not surprising that the predictive accuracy for recurrence was among the best when PC1 of all the MRI metrics was added to the clinicopathological variables of RCB and age.

There are a few limitations in this study. One major limitation is the small sample size. As mentioned earlier, AUC estimates can be unstable in small samples [41]. This preliminary study was not designed to power reliable predictive modeling; rather, it is mainly an exploratory study of the potential for enhancing prediction of recurrence by adding MRI metrics to clinicopathological variables. Small sample size also poses more risk for over-fitting of the data. In fact, the calculated cv ROC AUC values were decidedly lower than the apparent ROC AUC values. In addition, receptor status-based breast cancer molecular subtypes were not predictive of recurrence vs. non-recurrence. The number of patients was not adequate to draw meaningful conclusions in comparing the subtypes for discriminating patients with and without recurrence. As such, the preliminary findings reported here reflect the average results from a general breast cancer population undergoing NACT. Secondly, the data sets used in this study had moderate class imbalance with N = 7 for the recurrence group and N = 40 for the non-recurrence group. ROC AUC is commonly used in this field as the metric for predictive performance. However, when there is a large class imbalance in the outcome variable, ROC curves can be deceptively optimistic. This can also cause challenges in comparing predictive models. ROC AUC of the clinicopathological variable-only model already provided an inflated view of performance with AUC = 0.900, which left little room for improvement when adding the MRI metrics. In such cases, Precision-Recall (PR) curves are often assessed as they can show differences between models that are not discernible in ROC analysis, and give a more informative presentation of the predictive performance. We explored the PR curves of the predictive models used in this study. However, they did not reveal more differences between the models than the ROC curves (data not shown). Thirdly, the mean tumor DCE-MRI PK parameter values were used in this study for correlations with the recurrence endpoint. It is well known that malignant tumors are heterogeneous in nature and responses to treatment are likely heterogeneous as well. However, the heterogeneity in breast tumor functional changes in response to NACT was not captured in computing mean DCE-MRI parameter values. Recent studies show that radiomics analysis of either raw image data [26, 43] or parametric maps of quantitative functional parameters [48, 49] can be a valuable tool for characterizing tumor heterogeneity. Measurement and integration of both mean values and texture features of DCE-MRI parameters through radiomics analysis may further improve the robustness of quantitative DCE-MRI for prediction of breast cancer recurrence following NACT.

In conclusion, the results from this preliminary study show that quantitative DCE-MRI parameters may outperform tumor size measurement in prediction of breast cancer recurrence following NACT, whether alone or in combination with clinicopathological variables. Combining a single DCE-MRI parameter of perfusion and permeability or PC1 of all MRI metrics with clinicopathological variables in a predictive model showed potential of added value of MRI for improving accuracy in prediction of recurrence. Accurate prediction of recurrence pre- and/or post-NACT may help improve clinical decision making in adjusting NACT and/or adjuvant treatment regimens to reduce the risk of recurrence and improve survival outcome. It is important to validate our preliminary findings from this small patient cohort with a larger patient population in the future and update the predictive models to account for differences in NACT and adjuvant therapy regimens among the patients.

## Data Availability

The data that support the findings of this study are available on request from the corresponding author. The data are not publicly available due to information that could compromise the privacy of research participants.
